# Advancing BiVO_4_ Photoanode Activity for Ethylene Glycol Oxidation via Strategic pH Control

**DOI:** 10.3390/molecules29122783

**Published:** 2024-06-11

**Authors:** Jun-Yuan Cui, Tian-Tian Li, Long Chen, Jian-Jun Wang

**Affiliations:** 1 State Key Laboratory of Crystal Materials, Shandong University, Jinan 250100, China; 202132830@mail.sdu.edu.cn (J.-Y.C.); litiantian@mail.sdu.edu.cn (T.-T.L.); 202232804@mail.sdu.edu.cn (L.C.); 2 Shenzhen Research Institute of Shandong University, Shenzhen 518057, China

**Keywords:** BiVO_4_ photoanode, ethylene glycol oxidation, pH control, photoelectrochemical

## Abstract

The photoelectrochemical (PEC) conversion of organic small molecules offers a dual benefit of synthesizing value-added chemicals and concurrently producing hydrogen (H_2_). Ethylene glycol, with its dual hydroxyl groups, stands out as a versatile organic substrate capable of yielding various C1 and C2 chemicals. In this study, we demonstrate that pH modulation markedly enhances the photocurrent of BiVO_4_ photoanodes, thus facilitating the efficient oxidation of ethylene glycol while simultaneously generating H_2_. Our findings reveal that in a pH = 1 ethylene glycol solution, the photocurrent density at 1.23 V vs. RHE can attain an impressive 7.1 mA cm^−2^, significantly surpassing the outputs in neutral and highly alkaline environments. The increase in photocurrent is attributed to the augmented adsorption of ethylene glycol on BiVO_4_ under acidic conditions, which in turn elevates the activity of the oxidation reaction, culminating in the maximal production of formic acid. This investigation sheds light on the pivotal role of electrolyte pH in the PEC oxidation process and underscores the potential of the PEC strategy for biomass valorization into value-added products alongside H_2_ fuel generation.

## 1. Introduction

The surge in global energy demand and growing environmental concerns are propelling the advancement of green energy and renewable chemicals [1,2,3,4,5]. Molecular hydrogen serves as both a fundamental building block for the chemical industry and a promising carbon-free energy carrier [6,7,8]. Over the past few decades, photoelectrochemical (PEC) water splitting has emerged as a viable method to harness solar energy and generate clean hydrogen fuel [9,10,11,12]. In recent decades, photoelectrochemical (PEC) water splitting has emerged as a promising method for harnessing solar energy to produce clean hydrogen fuel, addressing the challenges of solar energy intermittency [13]. Compared to photocatalysis, the key advantage of photoelectrocatalysis is its direct conversion of light energy into electrical energy, leading to enhanced catalytic efficiency. This enables better control over charge separation and transfer, ultimately improving the overall performance and selectivity of catalytic reactions. Understanding the working principles of PEC cells is crucial as researchers can optimize cell component properties to enhance PEC performance. When illuminated, the PEC goes through six processes: light absorption by the semiconductor film, the generation of electron–hole pairs, electron flow to the counter electrode for current production, reduction at the counter electrode/electrolyte interface, oxidation at the semiconductor/electrolyte interface, and the recombination of electrons and holes in the semiconductor film [14,15]. However, the sluggishness of the oxygen evolution reaction (OER) at the anode has been a major hurdle in PEC water splitting, leading to high energy consumption [16,17,18,19].

To address this challenge, it is proposed to replace the OER with the oxidation of small molecules with lower oxidation potentials [20]. This strategy promises higher energy efficiencies and greater current density. Polyethylene terephthalate (PET), a widely used plastic, is a prime candidate for degradation and recycling due to its versatile properties [21,22]. Ethylene glycol, a hydrolysis byproduct of PET, is particularly notable for its annual production volume and favorable properties, including low toxicity and high energy density [23,24,25,26]. The selective oxidation of biomasses such as ethylene glycol could lead to the production of valuable compounds like glycolic acid, formic acid, and oxalic acid [25,27]. Integrating biomass oxidation with hydrogen evolution reactions could enhance current output at lower potentials, potentially reducing issues like photocorrosion [28,29]. Repurposing discarded materials like ethylene glycol without relying on fossil fuels can significantly contribute to sustainability efforts. Thus, the rational usage of ethylene glycol is highly desirable but still challenging.

Among the various photoanodes, BiVO_4_ stands out for its cost effectiveness, narrow bandgap enabling suitable light absorption, and high activity [30,31,32]. It has found widespread application in the PEC oxidation of biomass, coupled with hydrogen production. While BiVO_4_ has been extensively studied for PEC glycerol oxidation [33,34,35,36], studies on other alcohols are limited. For example, Liu et al. explored the impact of pH on PEC glycerol oxidation, finding that glycerol adheres better to BiVO_4_ at lower pH levels, facilitating charge transfer and catalyzing the conversion of glycerol into derivatives under photoelectrochemical conditions [36]. The pH of the electrolyte has emerged as a crucial factor influencing PEC performance in glycerol oxidation [37], with acidic electrolytes promoting the oxidation process. To broaden the scope of its applications to other alcohols, understanding the effect of electrolyte pH on the PEC ethylene glycol oxidation of BiVO_4_ is essential yet unexplored. Here, we systematically investigate the influence of electrolyte pH on the PEC performance for ethylene glycol oxidation and elucidate its underlying mechanism.

## 2. Results and Discussion

### 2.1. Synthesis and Structural Characterizations of the BiVO_4_ Photoanode

**Figure 1:**
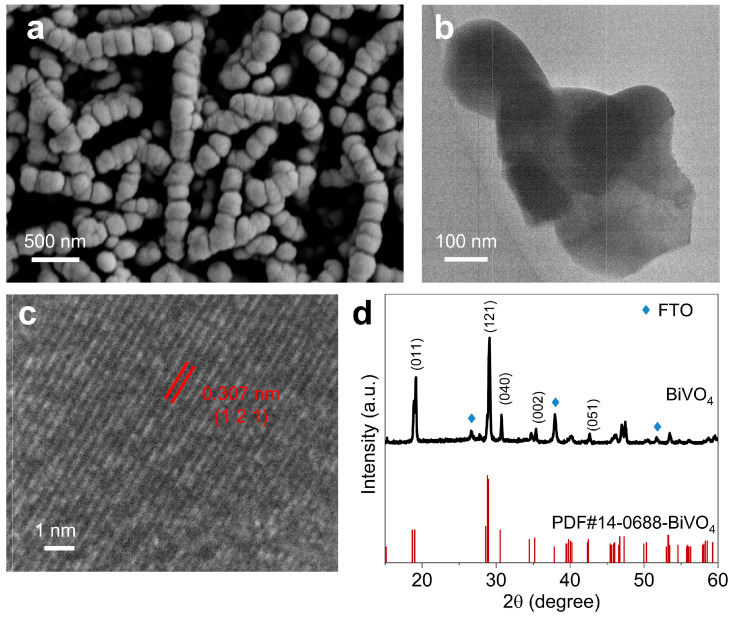
(**a**) SEM, (**b**) TEM, and (**c**) HRTEM images and (**d**) the XRD pattern of the as-prepared BiVO_4_.

Nanoporous BiVO_4_ films were synthesized with minor adjustments to a previously established protocol [38]. Initially, BiOI nanoflake arrays (Figure S1) were electrodeposited on fluorine-doped tin dioxide (FTO). Subsequently, the BiVO_4_ photoanode was obtained through further annealing with vanadyl acetylacetonate at elevated temperatures. The scanning electron microscopy (SEM) image in Figure 1a depicts the as-prepared BiVO_4_ photoanode, showcasing a typical nanorod array structure with an average diameter of approximately 200 nm, similar to the related reports on BiVO_4_ [39]. Similarly, the transmission electron microscopy (TEM) image (Figure 1b) reveals irregular and adhesive nanoparticles (≈200 nm) constituting the morphology of the BiVO_4_ photoanode. The high-resolution TEM (HRTEM) image (Figure 1c) demonstrates a lattice distance of 0.307 nm, consistent with the spacing of the (121) plane of monoclinic BiVO_4_ (JCPDS#14-0688), confirming successful synthesis of BiVO_4_ [38]. The X-ray diffraction (XRD) pattern of the sample (Figure 1d) indicates the absence of characteristic peaks of vanadium oxides, with all diffraction peaks assignable to monoclinic BiVO_4_ (JCPDS#14-0688), further confirming the crystal structure of BiVO_4_ without any impurities. Additionally, the optical properties of the prepared BiVO_4_ photoanode were examined via the UV–vis diffuse reflectance spectrum (DRS), revealing an absorption edge at approximately 500 nm (Figure S2), resulting in a bandgap of 2.54 eV according to the Tauc plot for the direct bandgap of BiVO_4_ (inset of Figure S2) [40].

### 2.2. Photoelectrochemical Performance of the BiVO_4_ Photoanode

**Figure 2:**
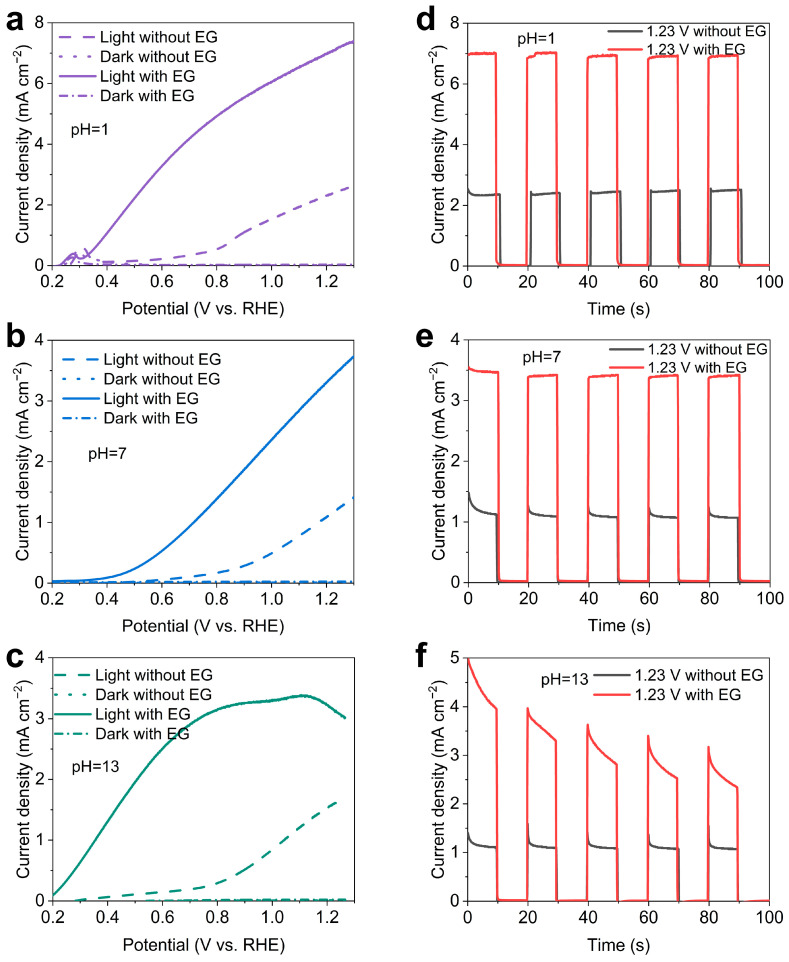
The PEC performance measured in various pH electrolytes with and without ethylene glycol. (**a**–**c**) Current density–potential profiles of the BiVO_4_ photoanodes under dark and light illumination. (**d**–**f**) Chopped photocurrent density–time profiles of the BiVO_4_ photoanodes at 1.23 V vs. RHE.

The PEC performance of the BiVO_4_ photoanode was evaluated in electrolytes with various pH values (1, 7, 13) under one sun illumination (AM 1.5 G, 100 mW cm^−2^). A three-electrode setup within a quartz cell, employing Pt foil as the counter electrode and Ag/AgCl or Hg/HgO as the reference electrode, was utilized. Figure 2 illustrates the current density–potential profiles under dark and illumination conditions. In the absence of ethylene glycol in the reaction medium, the photocurrent density resulting from water oxidation via back illumination shows maximal variation. Notably, the highest photocurrent density occurs in pH = 1 electrolyte (2.4 mA cm^−2^), while values at pH = 7 and 13 are 1.18 and 1.61 mA cm^−2^ at 1.23 V vs. RHE, respectively. The introduction of ethylene glycol leads to a significant increase in photocurrent density and a clear onset shift towards lower potentials, indicating easier oxidation of ethylene glycol than water [36,41]. Specifically, the photocurrent densities in pH = 7 and 13 reach 3.44 and 3.12 mA cm^−2^, respectively, while in pH = 1, the highest photocurrent density of 7.10 mA cm^−2^ at 1.23 V vs. RHE was achieved (Table S1). Apparently, the increase in pH decreases the photocurrent and increases onset potential, suggesting a direct influence of protons on the catalytic oxidation reaction. Additionally, with increasing the applied potential, the photocurrent density at pH = 13 experiences a decline, probably attributed to strongly alkaline-induced photocorrosion of BiVO_4_ [36]. The PEC performance in this study stands out significantly compared to recently reported counterparts like WO_3_/TiO_2_ and Ti–Fe_2_O_3_/Ni(OH)_x_ (Table S2).

Figure 2d–f illustrate the chopped photocurrent profiles recorded at 1.23 V vs. RHE. In the absence of ethylene glycol, the slow kinetics of the water oxidation reaction result in the diffusion and accumulation of photogenerated holes at the BiVO_4_ surface, leading to a transient spike at each on–off cycle. Overall, the transient spike at pH = 1 was weaker compared to pH = 7 and 13, suggesting easier transfer of photogenerated holes for water oxidation reactions at pH = 1, which therefore diminishes the chopped photocurrent spikes [41]. The addition of 0.5 M ethylene glycol not only significantly increases the photocurrent density but also reduces the photocurrent spike simultaneously. This observation indicates faster reaction kinetics for ethylene glycol oxidation than water oxidation. Moreover, an increase in reaction pH decreases the photocurrent density, possibly due to better ethylene glycol adsorption on BiVO_4_ at lower pH, similar to previous reports, as will be demonstrated in subsequent experiments [36]. The enhanced ethylene glycol adsorption on BiVO_4_ at lower pH facilitates the transfer of photogenerated holes for further oxidation reactions, thereby reducing the chopped photocurrent spikes. Additionally, the photocurrent density at pH = 13 with ethylene glycol experiences a rapid decline within seconds, highlighting the high susceptibility of BiVO_4_ to photocorrosion in alkaline environments.

**Figure 3:**
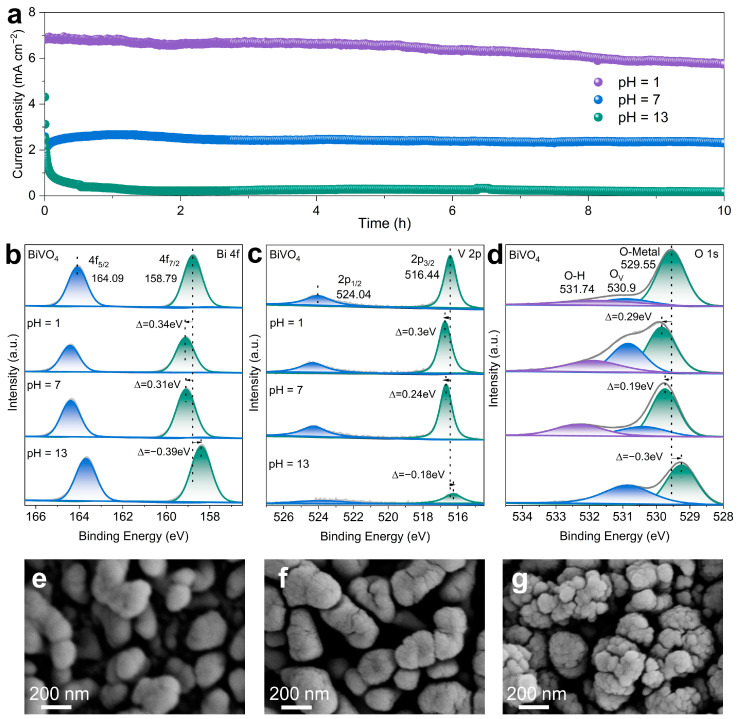
(**a**) Long-term stability of BiVO_4_ photoanode at 1.23 V vs. RHE in various pH with 0.5 M ethylene glycol under AM 1.5G, 100 mW cm^−2^ illumination. (**b**) Bi 4f, (**c**) V 2p, and (**d**) O 1s XPS peaks of the BiVO_4_ photoanode before and after the PEC tests in various pH electrolytes. SEM images of BiVO_4_ photoanodes after the PEC tests in (**e**) pH = 1, (**f**) pH = 7, and (**g**) pH = 13 with 0.5 M ethylene glycol.

Stability is also one of the most essential indicators for the practical application of a PEC system [42,43]. Therefore, the performance stability of PEC ethylene glycol oxidation was examined through current density–time curves at 1.23 V vs. RHE. As illustrated in Figure 3a, in the presence of ethylene glycol, the photocurrent density rapidly decreased for hundreds of seconds in pH = 13. However, in pH = 1 and 7, the photocurrent density maintained largely stability over an impressive 10 h span. Additionally, the photocurrent of BiVO_4_ in pH = 1 with ethylene glycol was consistently significantly higher than in pH = 7 and 13, suggesting that lower pH benefits the ethylene glycol oxidation activity [44]. X-ray photoelectron spectroscopy (XPS) measurements were further carried out to study the changes in chemical states of the elements in the BiVO_4_ photoanode before and after the PEC tests [45]. As presented in Figure 3b,c, the Bi 4f7/2, Bi 4f5/2, V 2p3/2, and V 2p1/2 XPS peaks of the BiVO_4_ photoanode after the PEC test in the pH = 13 electrolyte shift to lower binding energy compared to pristine BiVO_4_, indicating an increase in electron cloud density around Bi, V, and O atoms, probably due to the formation of oxygen vacancies [46,47,48], which led to the charge density of Bi and V being increased after the PEC test. More interestingly, the Bi 4f peaks (Figure 3b), V 2p (Figure 3c), and O 1s XPS peaks (Figure 3d) of BiVO_4_ after testing in pH = 1 and 7 moved to higher binding energy compared with pristine BiVO_4_, suggesting electron density reduction in the elements. These results imply an increase in the oxidized states of BiVO_4_ after ethylene glycol oxidation reaction [49,50]. Furthermore, the characteristic peaks of Bi element could be detected after the PEC stability tests, but their contents were slightly decreased compared with the pristine samples, which can be assigned to the photo-induced Bi^3+^ dissolution from the BiVO_4_ lattices [32]. Similarly, the peak intensity of V element was also decreased, indicating a large amount of V element dissolution, leading to a significant decrease in stability in the pH = 13 electrolyte. SEM images were also obtained before and after the PEC tests (Figure 3e–g). The SEM images of the BiVO_4_ photoanodes after the stability test in pH = 1 and 7 demonstrate that the nanoporous structure was slightly destroyed compared to the initial BiVO_4_ photoanode (Figure 1a). Surprisingly, the structure was completely destroyed after the PEC test in the pH = 13 electrolyte, which is consistent with the decay of the current density–time curve. In contrast, the XRD patterns were acquired after testing in pH = 1 and 13 (Figure S3), which illustrate that the photoanodes maintain their structures. However, the peak intensity was thoroughly weakened, which is consistent with the stability results.

### 2.3. Charge Transport and Dynamics in PEC Ethylene Glycol Oxidation

**Figure 4:**
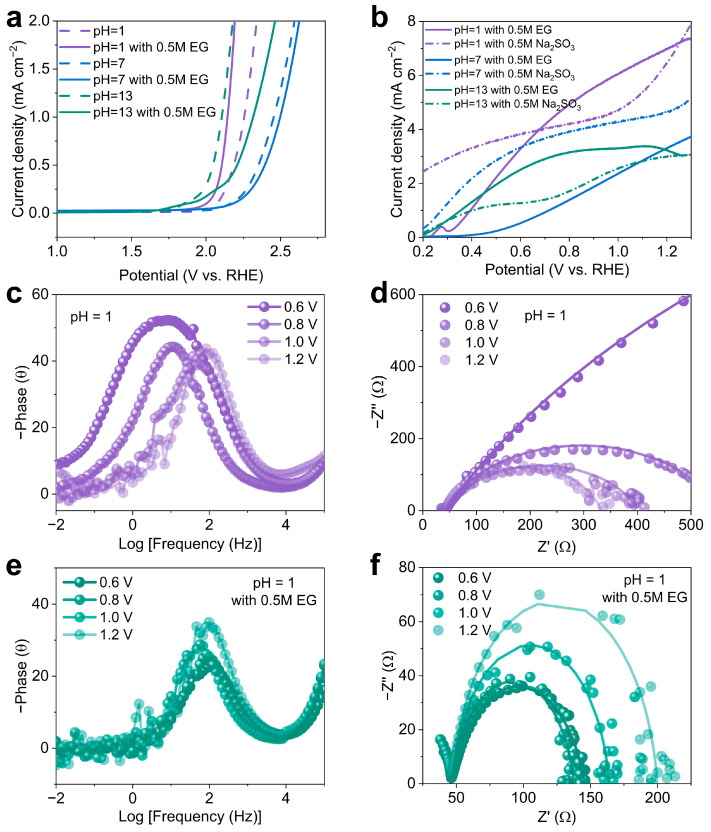
(**a**) LSV curves measured in the dark. (**b**) LSV curves obtained in electrolytes with variable pH, with and without ethylene glycol and Na_2_SO_3_. Bode plots measured in different electrolytes at varying potentials (**c**) without and (**e**) with ethylene glycol. Nyquist plots recorded at distinct potentials in different electrolytes (**d**) without and (**f**) with ethylene glycol.

To elucidate the influence of pH on the oxidation kinetics of the BiVO_4_ photoanode, the overpotentials of the BiVO_4_ photoanodes for ethylene glycol oxidation were recorded in the dark with differing pH levels, as depicted in Figure 4a [44]. In the absence of ethylene glycol, the minimum overpotential was observed in the electrolyte with pH = 7, signifying the optimal catalytic activity at this pH. In contrast, the most pronounced increase in current density for ethylene glycol oxidation in the electrolyte with pH = 1 was noted in comparison to the pH = 7 and 13 environments, suggesting the enhanced electrocatalytic activity due to the accelerated reaction kinetics under low pH conditions. Commonly, Na_2_SO_3_ was used as a hole scavenger to evaluate the charge injection efficiency on the surface of the photoanodes [38]. As demonstrated in Figure 4b, the addition of Na_2_SO_3_ significantly enhanced the PEC oxidation current density, and the current density for the ethylene glycol oxidation reaction consistently outperformed that of the sulfite oxidation in pH = 1 and pH = 13 electrolytes within the potential range of 0.68–1.25 and 0.32–1.26 V vs. RHE, respectively, in which BiVO_4_ exhibits faster ethylene glycol oxidation kinetics than that of Na_2_SO_3_ [51]. This may suggest that the high current density was not only attributed to the photoelectric conversion of the BiVO_4_ photoanode, but also to an additional current from the photo-driven ethylene glycol oxidation process [40].

Electrochemical impedance spectroscopy (EIS) was employed to probe the charge and mass transfer processes under varying applied potentials under illumination and the results were fitted by an equivalent circuit model (Figure S4) [52]. The EIS spectra without ethylene glycol (Figure 4c,d) reveal a frequency peak decrease and shift towards higher frequencies and a decrease in the impedance semicircle diameter as bias increases, indicative of reduced faradaic resistance and an accelerated surface reaction rate [53]. Upon the introduction of ethylene glycol (Figure 4e,f), the frequency peak remains nearly unchanged and the impedance semicircle expands with increasing bias, signifying that the intensified ethylene glycol oxidation process hampers the rapid desorption of reaction intermediates, thereby elevating the resistance, consistent with previous reports [36,54]. In order to examine alterations in surface states, Mott–Schottky plots were generated under light conditions both with and without EG (Figure S5). The findings revealed a subtle shift towards lower potentials on the Mott–Schottky plot following the introduction of EG. This shift implies that surface-state charging, specifically hole accumulation, is significantly inhibited, highlighting the enhanced kinetics of EG oxidation compared to water.

### 2.4. Product Analysis and Reaction Mechanism of PEC Ethylene Glycol Oxidation

**Figure 5:**
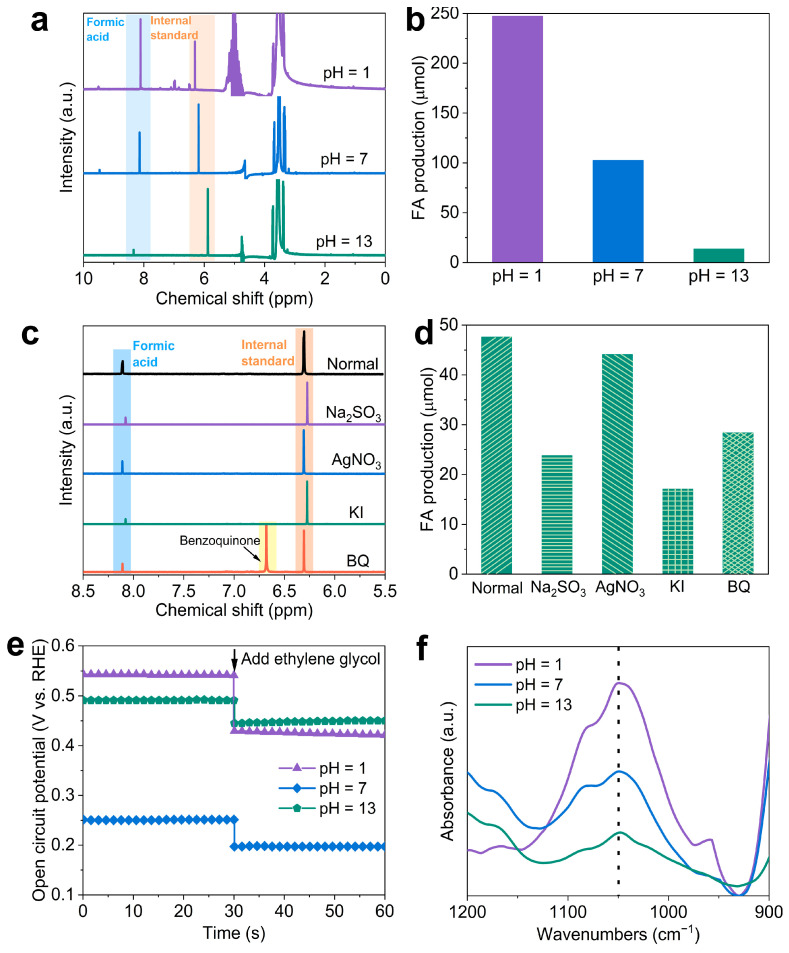
(**a**) ^1^H NMR spectra (400 MHz, D_2_O) of formic acid after 10 h ethylene glycol oxidation at 1.23 V vs. RHE on BiVO_4_ in various electrolytes. (**b**) FA production in various electrolytes with ethylene glycol. (**c**) ^1^H NMR spectra (400 MHz, D_2_O) of formic acid after 2 h ethylene glycol oxidation at 1.23 V vs. RHE on BiVO_4_ in the presence of various radical scavengers. (**d**) FA production in the presence of various radical scavengers (5 mM) for 2 h (Normal, no scavenger; Na_2_SO_3_ as the hole scavenger, AgNO_3_ as the electron scavenger, KI as the •OH radical scavenger, and BQ as the superoxide anion radicals). (**e**) Variation of open-circuit voltage in different pH conditions. (**f**) ATR-FTIR spectra of the BiVO_4_ photoanodes after immersion in different electrolytes.

The oxidation products of ethylene glycol over BiVO_4_ at 1.23 V vs. RHE in various pH electrolytes containing 0.5 M ethylene glycol were analyzed under AM 1.5G illumination (100 mW cm^−2^) over a period of 10 h [51,55]. The ^1^H NMR spectra (Figure 5a) clearly show the presence of formic acid, the internal standard (maleic acid), H_2_O, and ethylene glycol at 8.2, 6.0, 4.9, and 3.5 ppm, respectively [28,56]. Comparative analysis of the ^1^H NMR spectra from electrolytes after 10 h of stability testing at 1.23 V revealed selective oxidation of ethylene glycol to formic acid (Figure 5a,b and Table S1) with the Faradic efficiency higher than 60%. The highest yield of formic acid was achieved in the electrolytes with pH = 1, suggesting that a strongly acidic environment favors the oxidation of ethylene glycol to formic acid.

To understand the PEC oxidation mechanism, radical quenching experiments were conducted [57,58]. As depicted in Figure 5c,d, the use of Na_2_SO_3_ as a hole scavenger nearly halted formic acid (FA) production, underscoring the crucial role of photogenerated holes in the oxidation of ethylene glycol. The addition of the electron scavenger AgNO_3_ displayed minimal effect on FA yield. Additionally, when potassium iodide (KI) was employed to quench •OH radicals, there was a notable reduction in FA yield. In contrast, trapping superoxide anion radicals (•O_2_^−^) with benzoquinone (BQ) caused a significant reduction in FA production. These findings indicate that the photogenerated hole and •OH and superoxide anion radicals are involved in the PEC oxidation of ethylene glycol.

The adsorption of ethylene glycol species plays a critical role in the electrocatalytic oxidation process in the electrolyte [59]. The open-circuit voltage, which is influenced by the adsorption species in the Helmholtz layer [60], reflects the adsorption behavior of ethylene glycol on BiVO_4_ under different pH conditions. The open circuit potential (OCP) measurements (Figure 5e) demonstrate the effect of organic adsorbates on the inner Helmholtz layer [61]. Considering that the redox potential of ethylene glycol oxidation is lower than that of water, the contact between BiVO_4_ and the electrolyte results in a reduced equilibrium potential through electron transfer with the ethylene glycol-containing electrolyte. The addition of ethylene glycol significantly lowers the OCP in pH = 1 by 120 mV compared to pH = 7 (Δ = 54 mV) and pH = 13 (Δ = 41 mV), indicating more favorable adsorption of ethylene glycol on BiVO_4_ at pH = 1 [62]. This trend suggests that increasing pH leads to higher OH^−^ concentrations, which in turn decreases ethylene glycol adsorption. Further confirmation of ethylene glycol adsorption behavior was obtained through attenuated total reflectance Fourier-transform infrared spectroscopy (ATR-FTIR) tests (Figure 5f). The results of ATR-FTIR spectroscopy revealed a new peak at 1050 cm^−1^ [63] corresponding to the stretching vibrational adsorption peak of the C−O bond on BiVO_4_ after exposure to ethylene glycol solutions at varying pH levels. The highest peak intensity was observed after immersion in pH = 1, indicating enhanced adsorption of ethylene glycol onto the BiVO_4_ surface under acidic conditions. The point of zero charge (pH_PZC_) is a critical parameter for assessing the potential interactions between the surface charge of a catalyst and an organic compound. Below the pH_PZC_, the surface charge is positive, while it becomes predominantly negative at pH levels above the pH_PZC_. Interactions between nanoparticles and organic compounds occur when the nanoparticles are positively charged and the organic compound is negatively charged, or vice versa. The pH_PZC_ of BiVO_4_ has been reported to be approximately 4.2 [64,65], indicating that in a pH = 1 electrolyte, the surface charge of BiVO_4_ is positive, which is advantageous for the adsorption and oxidation of ethylene glycol. These aforementioned results collectively confirm that the ethylene glycol adsorption on the BiVO_4_ surface is most favorable at pH = 1, thereby maximizing the activity of the ethylene glycol oxidation reaction at this pH level.

## 3. Materials and Methods

### 3.1. Chemicals and Materials

Bismuth nitrate pentahydrate (Bi(NO_3_)_3_·5H_2_O, analytical reagent grade), p-benzoquinone (≥98.0%), and vanadyl acetylacetonate (VO(acac)_2_) were obtained from Sigma-Aldrich, Co., Ltd. (Burlington, MA, USA). Potassium iodide (KI, analytical reagent grade), dimethyl sulfoxide (DMSO, analytical reagent grade), glycerol (C_3_H_8_O_3_, analytical reagent grade), ethylene glycol (C_2_H_6_O_2_, analytical reagent grade), and nitric acid (HNO_3_) were supplied by Sinopharm Chemical Reagent Co., Ltd. in Shanghai of China. Fluorine-doped tin oxide (FTO, dimensions 10 × 30 × 2.2 mm, resistance 17 Ω) glass substrates were sourced from Luoyang Guluo Glass Technology Co., Ltd. in Luoyang of China. All chemicals were of analytical grade and were used as received without any further purification.

### 3.2. Preparation of the BiVO_4_ Photoanode

The BiVO_4_ thin films were prepared using a modified method previously described by Choi [38], which involves electrodeposition followed by air annealing. Initially, 0.97 g of Bi(NO_3_)_3_·5H_2_O was dissolved in 50 mL of 0.4 M KI solution, and the pH was adjusted to 1.7 using HNO_3_ to prepare solution A. Subsequently, 0.4972 g of p-benzoquinone (0.23 M) was dissolved in 20 mL of anhydrous ethanol to form solution B. Solution B was then gradually added to solution A under vigorous stirring for 30 min at room temperature, resulting in solution C. The BiOI films were fabricated through electrodeposition using a three-electrode system, with the FTO glass serving as the working electrode, a Pt plate as the counter electrode, and a Ag/AgCl electrode as the reference electrode. Solution C was used as the electrolyte. Electrodeposition was carried out at a constant potential of −0.1 V vs. Ag/AgCl for 540 s. The obtained orange BiOI precursors were washed with deionized water, dried with N_2_, and subsequently coated with a DMSO solution containing 0.2 M VO(acac)_2_, referred to as solution D. To finalize the BiVO_4_ photoanodes, 0.11 mL of solution D was applied to each BiOI electrode (1 × 2 cm^2^). The assemblies were then heated in air at a rate of 2 °C/min to 450 °C and held at this temperature for 120 min. After natural cooling, the obtained samples were immersed in 1 M NaOH solution for 30 min with gentle stirring to eliminate any residual vanadium oxide. The final BiVO_4_ film electrodes were thus obtained.

### 3.3. Characterization

The crystalline structures of the samples were analyzed using X-ray diffraction (XRD, Bruker D2 Phaser, Bruker, Billerica, MA, USA) and Raman spectroscopy (HORIBA XploRA Nano, Horiba, Kyoto, Japan). The morphology and structural details were investigated via field emission scanning electron microscopy (SEM, HITACHI S-4800, HITACHI, Tokyo, Japan) and transmission electron microscopy (TEM, JEOL JEM-2100F, Jeol Jem, Tokyo, Japan). The optical properties were assessed through UV-Vis-IR spectrophotometry (UV-2600, Bruker, Billerica, MA, USA) equipped with an integrating sphere. Surface compositions and electronic states were studied using X-ray photoelectron spectroscopy (XPS, X Per3 Powder, Bruker, Billerica, MA, USA). Proton nuclear magnetic resonance (^1^H NMR) spectra were recorded on a Bruker Advance III HD400 spectrometer, Bruker, Billerica, MA, USA.

### 3.4. Photoelectrochemical Measurements

The PEC experiments were conducted using a CHI660E electrochemical workstation (Shanghai Chen Hua Instruments Co., Shanghai, China) in a three-electrode configuration. The working electrode was the as-prepared BiVO_4_ photoanode with an area of 1 × 1 cm^2^, illuminated from behind. The reference and counter electrodes were a Ag/AgCl electrode and a 1 × 1 cm^2^ Pt sheet, respectively. The electrolytes used were (1) 0.1 M HNO_3_ acidic solution, with or without ethylene glycol, at pH = 1; (2) a neutral solution adjusted to pH = 7 using 1 M NaOH and 0.1 M HNO_3_; and (3) a 0.1 M NaOH alkaline solution at pH = 13. The light source was a 300 W xenon lamp (PLS-SXE300D; the light irradiation spectrum is shown in Figure S6) equipped with an AM 1.5G filter, set to an intensity of 100 mW cm^−2^. Linear sweep voltammetry (LSV) curves were recorded from 0.2 to 1.3 V vs. RHE at a scan rate of 10 mV/s, both with and without 0.5 M ethylene glycol. The impedance spectra were obtained from 0.01 to 10^5^ Hz with a 5 mV amplitude under AM 1.5G illumination, at potentials ranging from 0.6 V to 1.2 V vs. RHE. A stability test for the BiVO_4_ photoanodes in the presence of ethylene glycol was conducted across various pH electrolytes with 0.5 M ethylene glycol at 1.23 V vs. RHE under AM 1.5G illumination for 10 h. The applied potentials vs. Ag/AgCl and Hg/HgO were converted to the RHE scale using the Nernst equation:

E_RHE_ = E_Ag/AgCl_ + 0.059 pH + 0.1972

E_RHE_ = E_Hg/HgO_ + 0.059 pH + 0.098

Radical quenching experiments were conducted using BiVO_4_, incorporating various radical scavengers at a concentration of 5 mM over a 2 h period. The specific scavengers included KI for hydroxyl radicals, Na_2_SO_3_ as a hole scavenger, AgNO_3_ as an electron scavenger, and BQ for superoxide radicals, while a control sample was carried out without any scavengers. These experiments were carried out in a 0.1 M HNO_3_ electrolyte at pH = 1, with 0.5 M ethylene glycol, under AM 1.5G illumination at an intensity of 100 mW cm^–2^.

### 3.5. The Product Analysis

To quantitatively assess the products, ^1^H NMR data were acquired using an Advance III HD400 spectrometer (400 MHz). Initially, the electrolyte was collected after the stability test at an applied voltage of 1.23 V vs. RHE for 10 h for ethylene glycol oxidation under AM 1.5G light at 100 mW cm^−2^. Then, 500 µL of this electrolyte was transferred to an NMR tube, to which 40 µL of a 20 mM maleic acid solution and 100 µL of deuterium water (D_2_O) were added. The mixture was sonicated briefly to ensure homogeneity. The resultant ^1^H NMR spectrum revealed peaks for ethylene glycol, maleic acid, and D_2_O at 3.5, 5.9, and 4.8 ppm, respectively. All NMR data were analyzed using MestReNova 11.0 software.

## 4. Conclusions

In summary, our research underscores the profound influence of electrolyte pH on the PEC oxidation of ethylene glycol using BiVO_4_, optimizing photocurrent output. In a pH = 1 electrolyte, we observed an enhancement in charge injection efficiency, leading to a superior photocurrent density of 7.1 mA cm^−2^ at 1.23 V vs. RHE and the highest yield of formic acid, compared to neutral and alkaline conditions (pH = 7 and pH = 13). The comprehensive experimental analysis confirms that the superior adsorption properties of BiVO_4_ on ethylene glycol under acidic conditions are a key factor in the increased oxidation reaction activity. The mechanistic investigation of the PEC process indicates the involvement of multiple reaction pathways in the oxidation of ethylene glycol. This study not only highlights the critical impact of pH in modulating PEC biomass oxidation but also accentuates the promising potential of the PEC approach in the sustainable synthesis of valuable chemicals and energy carriers.

## Data Availability

Data will be made available on request.

## Supplementary Materials

The following supporting information can be downloaded at https://www.mdpi.com/article/10.3390/molecules29122783/s1: Figure S1: A top-view SEM image of the BiOI nanoflake array; Figure S2: The UV–vis DRS with the Tauc plot of the BiVO_4_ film. Figure S3: XRD patterns of the BiVO_4_ photoanodes after testing in various electrolytes in the presence of ethylene glycol. Figure S4: The equivalent circuit model for EIS analysis. Figure S5: Mott–Schottky plots of the BiVO_4_ photoanode in pH = 1 electrolyte with and without ethylene glycol. Figure S6: The standard reflection spectrum of the used light source. Table S1: The PEC performance in different pH electrolytes with 0.5 M ethylene glycol at 1.23 V vs. RHE. Table S2: Comparison of the results in this work with the reported literature on PEC ethylene glycol oxidation, References [66,67].
