# Supplementary material for: Advancing BiVO4 Photoanode Activity for Ethylene Glycol Oxidation via Strategic pH Control
Source: Molecules. 2024 Jun 11;29(12):2783. doi: 10.3390/molecules29122783 (PMC11206287; doi:10.3390/molecules29122783)
Supplement: Supplementary file 1 [file molecules-29-02783-s001.zip › molecules-3036171-supplementary.pdf]

Supplementary Materials

Advancing $BiVO_4$ Photoanode Activity for Ethylene Glycol Oxidation via Strategic pH Control

Jun-Yuan Cui [1], Tian-Tian Li [1], Long Chen [1] and Jian-Jun Wang [1,2,*]

[1] State Key Laboratory of Crystal Materials, Shandong University, Jinan 250100, China
[2] Shenzhen Research Institute of Shandong University, Shenzhen 518057, China
[*] Correspondence: wangjianjun@sdu.edu.cn

The supplementary figures

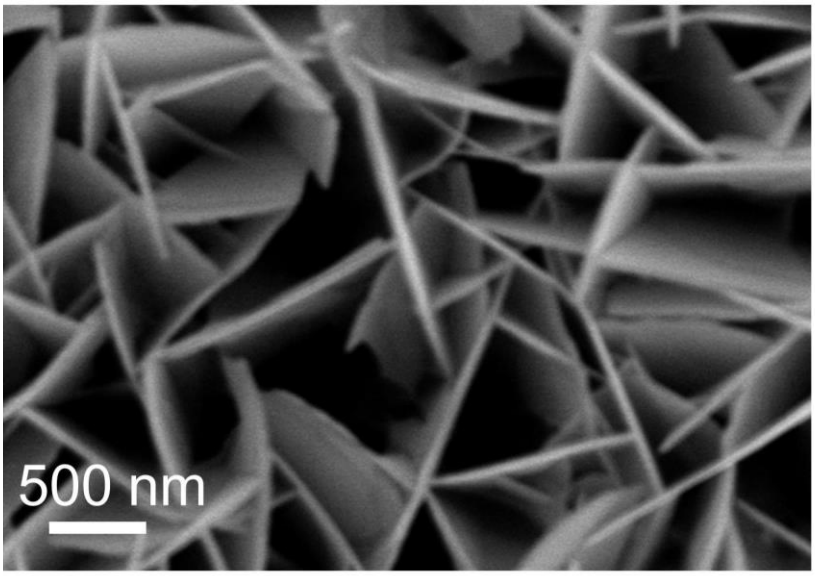

Figure S1. A Top-view SEM image of the BiOI nanoflake array.

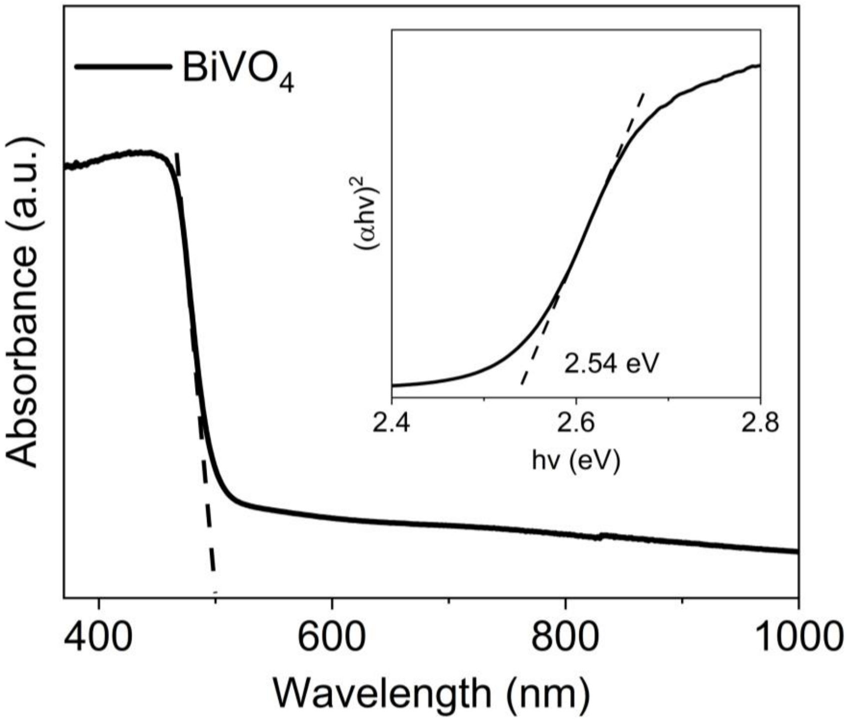

Figure S2. The UV–vis DRS spectrum with the Tauc-plot of the $BiVO_4$ film.

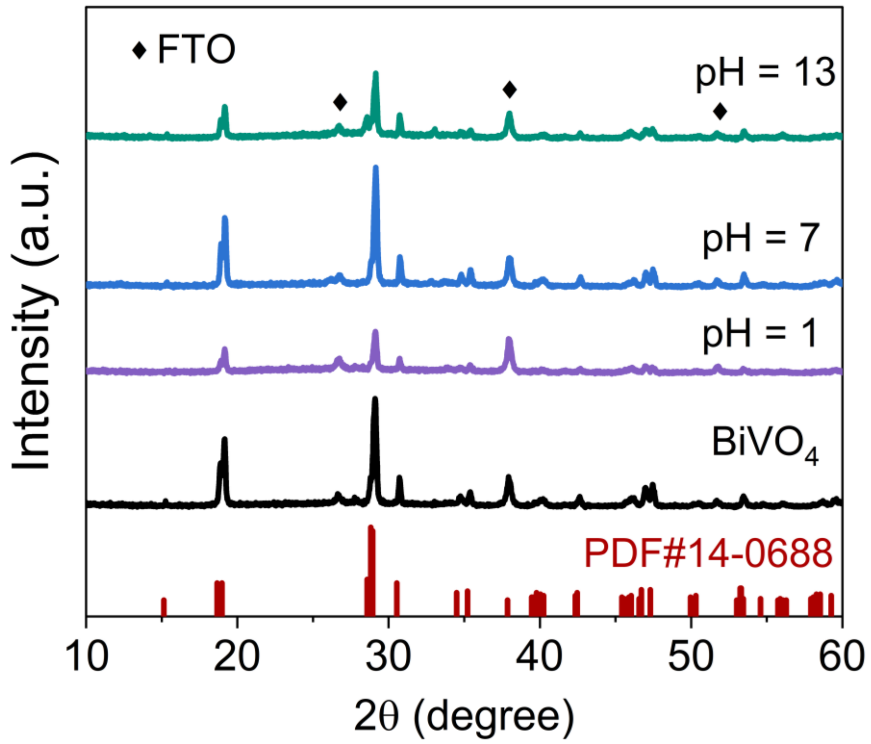

Figure S3. XRD patterns of the $BiVO_4$ photoanodes after testing in various electrolytes in the presence of ethylene glycol.

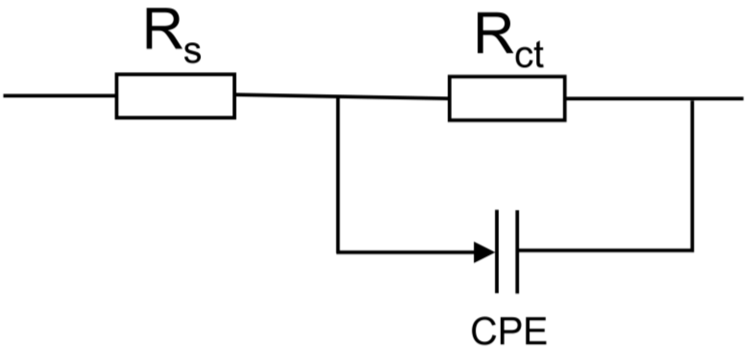

Figure S4. The equivalent circuit model for EIS analysis.

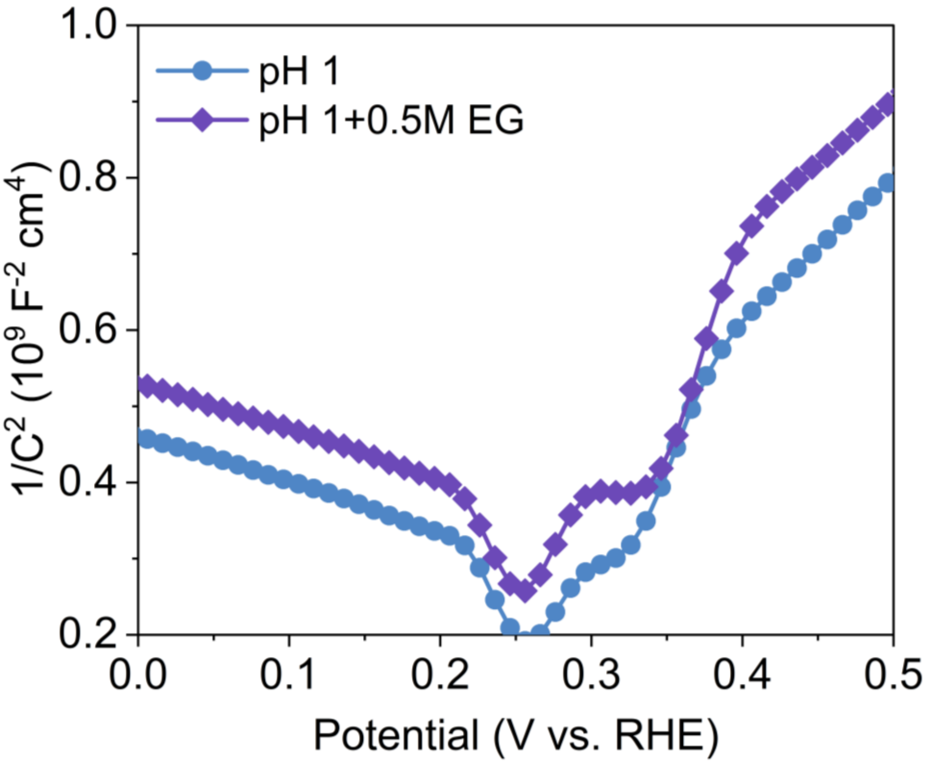

Figure S5. Mott–Schottky plots of the $BiVO_4$ photoanode in pH=1 electrolyte with and without ethylene glycol.

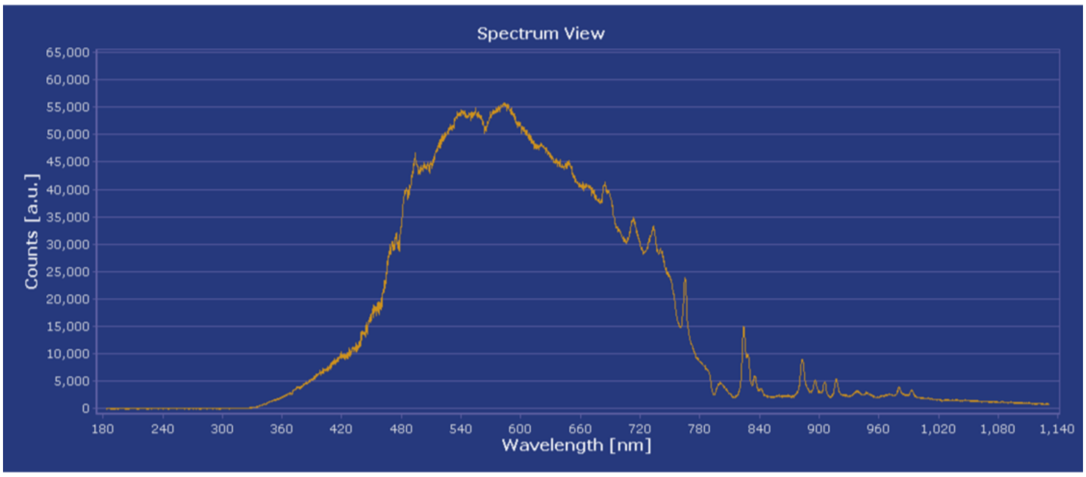

Figure S6. The standard reflection spectrum of the used light source.

Table S1. The PEC performance in different pH electrolytes with 0.5 M ethylene glycol at 1.23 V vs. RHE.

Electrolyte	pH=1	pH=7	pH=13
Photocurrent density ($mA\ cm^{-2}$)	7.10	3.44	3.12
FA production rate ($\mu mol\ cm^{-2}\ h^{-1}$)	24.73	10.25	1.37
Faradic efficiency (%)	62.02	67.81	79.87

Table S2. Comparison of the results in this work with the reported literature on PEC ethylene glycol oxidation.

Photoanode	Electrolyte	Photocurrent density ($mA\ cm^{-2}$)	Reference
**$BiVO_4$**	**0.1M HNO_3**	**7.1 (1.23 V vs. RHE)**	**This work**
WO_3/TiO_2	1 M KOH	0.07 (0.6 V vs. Ag/AgCl)	[1]
Ti–$Fe_2O_3/Ni(OH)_x$	1 M KOH	3 (1.2 V vs. RHE)	[2]

Reference

1. Mohammadnezhad, G.; Momeni, M.M.; Nasiriani, F., Enhanced photoelectrochemical performance of tin oxide decorated tungsten oxide doped TiO_2 nanotube by electrodeposition for water splitting. *J. Electroanal. Chem* **2020**, *876*, 114505.
2. Li, X.; Wang, J.; Sun, M.; Qian, X.; Zhao, Y., Ti–Fe_2O_3/$Ni(OH)_x$ as an efficient and durable photoanode for the photoelectrochemical catalysis of PET plastic to formic acid. *Journal of Energy Chemistry* **2023**, *78,* 487-496.
